# Pembrolizumab-Induced Insulin-Dependent Diabetes Mellitus in a Patient With Triple-Negative Breast Cancer: A Rare Immune-Related Adverse Event

**DOI:** 10.7759/cureus.73049

**Published:** 2024-11-05

**Authors:** Mirac Burak Tak, Zaid Munir, Ahmet Aydin

**Affiliations:** 1 General Medicine, University Hospitals Sussex National Health Service (NHS) Foundation Trust, Worthing, GBR; 2 Internal Medicine, Hospital Corporation of America (HCA) Brandon Hospital, Brandon, USA

**Keywords:** diabetes, immune checkpoint inhibitors, immune-related adverse event (irae), immune related adverse events, immunotherapy, immunotherapy induced diabetes, pd-1 inhibitor, pembrolizumab

## Abstract

A 68-year-old female patient with a background of triple-negative breast carcinoma on pembrolizumab with no history of diabetes presented to the emergency department with fatigue, polyuria, nausea, dizziness, shortness of breath, dry mouth, and increased thirst. She had recently received the third dose of the second cycle of neoadjuvant combination chemotherapy and immunotherapy (pembrolizumab/carboplatin/paclitaxel) and was due to receive the next dose. Initial assessment revealed hyperglycemia with ketosis without acidosis. The patient was treated with fluid resuscitation and insulin infusion under the diabetic ketoacidosis (DKA) guidelines of the hospital and was eventually transitioned to a basal-bolus insulin regimen, which was continued after discharge. Based on the temporal relationship between pembrolizumab therapy and the onset of diabetes, along with the patient's persistent insulin dependence, a diagnosis of immune checkpoint inhibitor-induced diabetes mellitus (ICI-DM) was established. The patient has clinically improved, chemotherapy and immunotherapy have been discontinued, and surgical intervention is planned. This case highlights the importance of recognizing ICI-DM as a rare immune-related adverse event in patients who receive immunotherapy with programmed cell death protein-1 (PD-1)/programmed cell death ligand-1 (PD-L1) inhibitors.

## Introduction

Pembrolizumab was granted Food and Drug Administration approval to be used in combination with chemotherapy for neoadjuvant treatment and as an adjuvant monotherapy following surgery in patients with high-risk early-stage triple-negative breast cancer [1]. The National Institute of Health and Care Excellence supports this and recommends its use in combination with chemotherapy for neoadjuvant treatment and as monotherapy for adjuvant therapy following surgery in adults with triple-negative early breast cancer at high risk of recurrence or locally advanced breast cancer [2].

PD-1 is a transmembrane receptor which regulates T-cell activation. When bonded with its ligand, it leads to T-cell apoptosis, a mechanism that physiologically prevents T cells from attacking healthy cells. However, cancer cells can exploit the PD-1 pathway, leading to T-cell exhaustion and immune evasion. Pembrolizumab is an immune checkpoint inhibitor (ICI) monoclonal antibody that targets PD-1, blocks this interaction, prevents T-cell exhaustion, and reactivates T cells. This mechanism, however, can lead to immune-related adverse events [3].

ICI-induced diabetes mellitus (ICI-DM) is a rare but significant adverse event [4]. With this case report, we aim to contribute to the growing body of evidence and raise awareness among clinicians.

## Case presentation

A 68-year-old female patient with a recent diagnosis of right breast carcinoma, identified as triple-negative grade 3 invasive mixed ductal and metaplastic carcinoma, presented to the emergency department with a two-day history of fatigue, shortness of breath, nausea, and dizziness. She had recently completed the third dose of the second cycle of combination chemotherapy and immunotherapy, which included pembrolizumab, carboplatin, and paclitaxel. The patient reported associated symptoms, including increased thirst, dry mouth, polyuria, reduced appetite, nausea, and generalized muscle aches.

The patient has no history of prediabetes or diabetes and no family history of diabetes. She has well-controlled hypertension, managed with lacidipine, perindopril, and indapamide. She is a lifelong non-smoker, and although she previously consumed alcohol moderately, she stopped drinking after her breast cancer diagnosis. She has no history of alcohol excess.

The patient was initially seen in the breast clinic with a six-week history of a right breast lump. On examination, a 3 cm suspicious mass was palpated in the upper outer quadrant of the right breast. Given her significant family history of breast cancer, malignancy was strongly suspected. Subsequent core biopsy confirmed a grade 3 invasive mixed ductal and metaplastic carcinoma. The tumor was further classified as triple-negative, and metastasis was ruled out. She was planned to undergo two cycles of neoadjuvant therapy with pembrolizumab, carboplatin, and paclitaxel. The first cycle, consisting of 12 weeks of combined chemotherapy and immunotherapy, was interrupted by missed doses at weeks 6 and 12 due to the patient feeling unwell. In the second cycle, only two doses were given before the patient became unwell.

Upon admission, the patient was afebrile, with a heart rate of 114 beats per minute, a blood pressure of 110/76 mmHg, a respiratory rate of 17 breaths per minute, and an oxygen saturation of 96% on room air. Her physical examination revealed drowsiness, dehydration, and mild epigastric discomfort. The rest of the physical examination was unremarkable. Her weight on admission was 115 kg, and her BMI was over 40.

The laboratory investigations revealed significant hyperglycemia with a blood glucose level of 32 mmol/L and an HbA1c of 111 mmol/mol (12.3%). The C-peptide level was 1554 pmol/L. Notably, tests for glutamic acid decarboxylase, zinc transporter 8, and islet antigen 2 antibodies were negative, indicating an absence of autoimmune markers commonly associated with type 1 diabetes.

Venous blood gas (VBG) analysis showed a pH of 7.37, pCO2 of 5.76 mmHg, lactic acid of 1.3 mmol/L, ketones of 5 mmol/L, and bicarbonate of 31.1 mmol/L, with an anion gap of 20.2 mmol/L. Urinalysis was positive for glucose (+4) and urinary ketones at 1.6 mmol/L. The urine C-peptide level was 21, with a C-peptide/creatinine ratio of 4.38. Importantly, HbA1c was 40 mmol/mol (5.8%), and random blood glucose was 6.8 mmol/L before initiating pembrolizumab therapy.

Adrenal function was assessed using the Synacthen test. A basal cortisol level of 890 nmol/L remained unchanged at 30 minutes post-Synacthen and rose to 1157 nmol/L at 60 minutes post-Synacthen.

Urinalysis was significant for glucosuria and ketonuria, blood tests showed mild hyponatremia and hypochloremia, serum amylase and other test results were normal (Table 1).

**Table 1:** Test results ALT, alanine aminotransferase; ALP, alkaline phosphatase; BNP, B-type natriuretic peptide; CK, creatine kinase; CRP, C-reactive protein; eGFR, estimated glomerular filtration rate; FSH, follicle-stimulating hormone; LH, luteinizing hormone; TSH, thyroid-stimulating hormone; T4, thyroxine (free T4); T3, triiodothyronine (free T3); B12, vitamin B12; µmol/L, micromole per liter; mmol/L, millimole per liter; ng/L, nanogram per liter; ng/mL, nanogram per milliliter; mIU/L, milli-international units per liter; U/L, units per liter; g/L, grams per liter; nmol/L, nanomole per liter; mg/L, milligrams per liter; IU/L, international units per liter; µg/L, microgram per liter; mL/min/1.73m², milliliters per minute per 1.73 square meters (for eGFR)

Blood test	Result	Reference range
Hemoglobin	126 g/L	120–160 g/L (female)
White cell count	5.5 x10^9/L	4.0–11.0 x10^9/L
Platelets	239 x10^9/L	150–450 x10^9/L
Sodium	129 mmol/L	135–145 mmol/L
Potassium	3.5 mmol/L	3.5–5.0 mmol/L
Chloride	90 mmol/L	98–107 mmol/L
Urea	7.2 mmol/L	2.5–7.8 mmol/L
Creatinine	59 µmol/L	45–84 µmol/L (female)
eGFR	90 mL/min/1.73 m²	>60 mL/min/1.73 m²
Albumin	33 g/L	35–50 g/L
ALT	45 U/L	7–56 U/L
ALP	79 U/L	30–120 U/L
Adjusted calcium	2.36 mmol/L	2.10–2.55 mmol/L
Magnesium	0.66 mmol/L	0.66–1.07 mmol/L
CRP	8 mg/L	<5 mg/L
Cortisol	273 nmol/L	200–700 nmol/L (morning)
Creatine kinase (CK)	31 U/L	30–200 U/L
BNP	<10 ng/L	<100 ng/L
Troponin I	8.2 ng/L	<17 ng/mL
Amylase	37 U/L	30–110 U/L
Vitamin B12	1011 ng/L	160–950 ng/L
Folate	7 µg/L	4.6–18.7 µg/L
FSH	26.8 IU/L	25.8–134.8 IU/L (postmenopausal)
LH	9.5 IU/L	7.7–58.5 IU/L (postmenopausal)
Testosterone	1.16 nmol/L	0.3–2.6 nmol/L (female)
Prolactin	193 mIU/L	102–496 mIU/L
TSH	0.55 mIU/L	0.4–4.0 mIU/L
Free T4	10.1 pmol/L	9.0–25.0 pmol/L
Free T3	3.19 pmol/L	3.1–6.8 pmol/L
Urine dipstick test	Result	Reference range
Glucose	>20 mmol/L	Negative
Ketones	1.6 mmol/L	Negative
pH	6.0	5.0-8.0
Protein	Negative	Negative
Nitrite	Negative	Negative
Leukocytes	Negative	Negative

ECG showed sinus rhythm with occasional premature ventricular complexes. A computed tomography pulmonary angiogram ruled out pulmonary embolism, and computed tomography of the abdomen showed no pancreatic abnormalities.

The patient was initially managed with the standard diabetic ketoacidosis (DKA) protocol, including intravenous fluids and a variable rate insulin infusion. Once the glycaemic control was achieved and ketosis resolved, she was transitioned to a basal-bolus regimen with subcutaneous insulin glargine. Given the patient's new insulin dependence, a diagnosis of ICI-DM was made.

The oncology team recommended discontinuing chemotherapy and immunotherapy, and surgical intervention is planned for the removal of carcinoma. The patient was discharged with a basal-bolus insulin regimen, including insulin glargine at 60 units once in the morning and insulin aspart, 12 units administered with each meal. Additionally, metformin 500 mg was prescribed twice daily. Currently, the patient is achieving good glycaemic control with this treatment.

## Discussion

Platinum-based chemotherapeutic agent cisplatin was rarely reported to cause T2DM and hyperglycaemic crisis; however, fewer cases are reported compared to immune checkpoint inhibitors, and these cases were not insulin-dependent. Carboplatin and paclitaxel combination have been reported to cause transient hyperglycemia in rats; however, these drugs are not commonly associated with the development of diabetes [5,6].

ICI-DM has been reported more since 2015, and the incidence ranges between 0.9% to 1.9% [7,8]. The onset of ICI-DM varies, occurring anywhere from weeks to over a year after starting therapy, with a reported median time to onset of 144.5 days [7-9].

The underlying mechanism of ICI-DM remains poorly understood. Animal studies have shown that blocking PD-1 and PD-L1 disrupts the immunoregulatory process, which can lead to autoimmune diabetes. Clinical studies reported a lower rate of PD-1 expression on helper T cells, which can cause increased cytotoxic T cell activity, leading to beta cell destruction [7].

The literature shows inconsistency regarding whether ICI-DM is linked to the presence of islet autoantibodies. Some research data showed that most patients are autoantibody-negative, while others show at least one islet autoantibody, with anti-glutamic acid decarboxylase being the most common [2]. Another theory suggests that rather than humoral immunity, cell-mediated immunity against islet cells plays a major role in autoantibody-negative type 1 diabetes in patients taking PD-1 inhibitor therapy [10]. Furthermore, it is uncertain whether autoantibodies are present before ICI therapy, making it unclear if they can reliably predict individuals at risk of ICI-DM [7].

Patients with ICI-DM tend to be older than those with type 1 diabetes mellitus and often require treatment in intensive care units [10].

Personal or family history of autoimmune conditions, presence of another immune-related adverse event, history of treatment with other immunotherapies, or using a combination of immunotherapies are risk factors for developing ICI-DM [7].

Monitoring blood glucose levels in patients undergoing treatment with immune checkpoint inhibitors is advised by the European Society for Medical Oncology [11]. American Society of Clinical Oncology suggests checking blood glucose every three to six weeks following the initial monitoring at baseline and with each treatment cycle for the first 12 weeks [12]. Insulin therapy is required for the vast majority of patients with ICI-DM, and this condition tends to be irreversible, necessitating lifelong insulin treatment [4,13]. Although some immune-related adverse events are treated with glucocorticoids, ICI-DM is shown to not respond to this treatment, and glucocorticoids may worsen hyperglycemia in these patients [14,15].

## Conclusions

Diabetes is a rare but dire adverse event of ICI therapy that can present as DKA or hyperglycemia without DKA. We presented a case of a 68-year-old female patient who presented with new-onset insulin-dependent diabetes after receiving pembrolizumab. Having a high level of suspicion for this immune-related adverse event, educating patients regarding the possible signs and symptoms, and checking blood glucose levels prior to starting immunotherapy and at regular intervals while continuing the therapy may help prevent complications and undesirable outcomes.

Further research into the underlying causes of this adverse event could help identify patients who are at higher risk and lead to better prevention strategies and treatment options.
